# Evaluation of modified Interferon alpha mRNA constructs for the treatment of non-melanoma skin cancer

**DOI:** 10.1038/s41598-018-31061-w

**Published:** 2018-08-28

**Authors:** Sarah Hochmann, Michaela Mittermeir, Radmila Santic, Frieder Koszik, Lanay Griessner, Alina Sarah Sonderegger, Thomas Hoffmann, Elisabeth Russe, Sandra Scheiblhofer, Richard Weiss, Markus Mandler, Achim Schneeberger, Dirk Strunk

**Affiliations:** 1 Cell Therapy Institute, Paracelsus Medical University, Salzburg, Austria; 2 Spinal Cord Injury and Tissue Regeneration Center Salzburg (Sci-TReCS), Paracelsus Medical University, Salzburg, Austria; 3 Accanis Biotech F&E GmbH & Co KG, Vienna Biocenter, Austria; 4 Department of Plastic, Aesthetic and Reconstructive Surgery, Hospital Barmherzige Brueder, Salzburg, Austria; 5 Department of Molecular Biology, Paris Lodron University, Salzburg, Austria

## Abstract

Application of *in vitro* transcribed (IVT) messenger ribonucleic acid (mRNA) is an increasingly popular strategy to transiently produce proteins as therapeutics in a tissue or organ of choice. Here, we focused on the skin and aimed to test if whole human skin tissue explant technology can be used to evaluate the expression efficacy of different IVT Interferon alpha (IFN-α) mRNA constructs *in situ*, after biolistic delivery. Skin explants were viable and intact for at least five days based on histologic analysis and TUNEL staining. Using GFP reporter mRNA formulations, we found mostly epidermal expression after biolistic delivery. Two out of five sequence-optimized IFN-α mRNA variants resulted in significantly improved IFN-α protein expression in human skin compared to native IFN-α mRNA transfection. IFN-α secretion analysis of the surrounding culture media confirmed these results. We provide a proof-of-concept that IFN-α mRNA delivery into intact human full thickness skin explants can be utilized to test mRNA sequence modifications *ex vivo*. This approach could be used to develop novel mRNA-based treatments of common epidermal skin conditions including non-melanoma skin cancer, where IFN-α protein therapy has previously shown a strong therapeutic effect.

## Introduction

Recent developments in nucleic acid-based therapeutics, especially ribonucleic acid (RNA)-based therapies, have shown significant potential for a wide range of human diseases (reviewed in^1^). Initial attempts towards *in vitro* transcribed (IVT) mRNA therapy date back to 1992, where a temporary reversal of diabetes insipidus in a rat model after intrahypothalamic injection of vasopressin mRNA was demonstrated^2^. However, clinical translation of this research for systemic diseases has been limited by enzymatic degradation of RNA molecules, low delivery to the diseased site, as well as toxicity concerns. In contrast, delivery of nucleic acids to the skin is advantageous because it is able to bypass many of these challenges associated with systemic administration, including simplifying application and increasing local bioavailabilty (reviewed in^3^), making it an ideal target for the development of RNA-based therapeutics.

Non-melanoma skin cancer (NMSC) is the most common form of cancer worldwide among people with fair complexions. NMSCs most notably include basal cell carcinoma (BCC), squamous cell carcinoma (SCC), and the *in situ* carcinoma actinic keratosis (AK). Ultraviolet light exposure is the greatest risk factor in the development of NMSCs. Despite growing public awareness of the harmful effects of excessive sun exposure, the incidence of NMSCs continues to rise^4^. The World Health Organization currently estimates that between two and three million NMSCs are diagnosed globally each year^5^.

Although surgical removal of NMSC lesions remains the gold-standard for treatment, it is not able to address the field-cancerization that is commonly associated with the condition, where a large surface area can be affected. To address the cancerous field, topical therapies such as imiquimod and ingenol mebutate are often used^6^. However, these treatments have the disadvantage of low patient compliance and are not recommended for high-risk lesions^7^.

Interferon alpha (IFN-α) is a key immunostimulatory molecule with potent anti-tumoral effects. Since the 1980s, perilesional treatment using IFN-α has been shown to be safe and effective against BCC^8–10^, SCC^11,12^, and AK^13,14^. However, its broad implementation in the clinic for NMSC therapy has been hindered for several reasons including the need of daily applications over 10–14 days as well as high costs of IFN-α.

Messenger RNA (mRNA) therapy provides one way to overcome these limitations. *In vitro* transcribed (IVT) mRNA therapy introduces mRNA transcripts into the cell cytoplasm to drive synthesis of a specific protein. Interest in the technology has surged in the past decade following the discovery of IVT mRNA modifications that reduced immunogenicity^15,16^ and increased stability^17,18^, making mRNA a viable therapeutic option also for protein replacement. Compared to DNA-based approaches, mRNA has the distinct advantage that it does not integrate into the host genome, minimizing potential treatment risks including insertional mutagenesis. Therapeutic protein expression efficiency following IVT mRNA application has been shown to be comparable to that obtained after plasmid DNA transfer^19^.

In this study, we explored the delivery of IFN-α IVT mRNA to the epidermis in human skin explants. To improve cellular bioavailability of the mRNA molecule and overcome the stratum corneum, we choose a biolistic approach that has been previously used to deliver DNA to both mouse^20–23^ and human skin^24^. Here, we demonstrate that this approach can successfully produce IFN-α expression in *ex vivo* human skin using IVT mRNA, and present optimized IFN-α mRNA sequence variants that show increased production compared to the native sequence. These results demonstrate the potential utility of an IFN-α IVT mRNA therapy for common epidermal skin conditions, including NMSCs, where IFN-α protein therapy has previously shown a strong therapeutic effect^25^.

## Results

### Establishment of skin tissue culture and biolistic delivery conditions

We first established conditions to keep skin viable for at least five days in culture. Punch biopsies of six compared to eight mm in diameter were obtained from whole thickness human skin within four hours following tissue collection after plastic reconstructive surgery (Fig. S1a,c). Punch biopsy derived skin explants were cultured at the air-liquid interface either in transwell inserts (Fig. S1d) or adherent to the tissue culture plastic in 150 cm^2^ tissue culture petri dishes (Fig. S1e,f). Histologic analysis in a time course after one, two and five days showed best preservation of skin appearance in 8 mm biopsies cultured at the air-liquid interface in petri dishes (Fig. S2). Viability was further tested by TdT-mediated dUTP-biotin nick end (TUNEL) labeling, which showed minimal signs of apoptosis after 5 days in culture (Fig. S2o–r). To functionally confirm the viability of cultured explants, fibroblast outgrowth was determined at the end of a five day explant culture. Radial outgrowth of primary fibroblasts was clearly visable around *ex vivo* cultures, indicating that the tissue remained functional (Fig. S3). We therefore continued testing 8 mm biopsies in subsequent experiments.

### eGFP mRNA is successfully expressed in the epidermis

**Figure 1:**
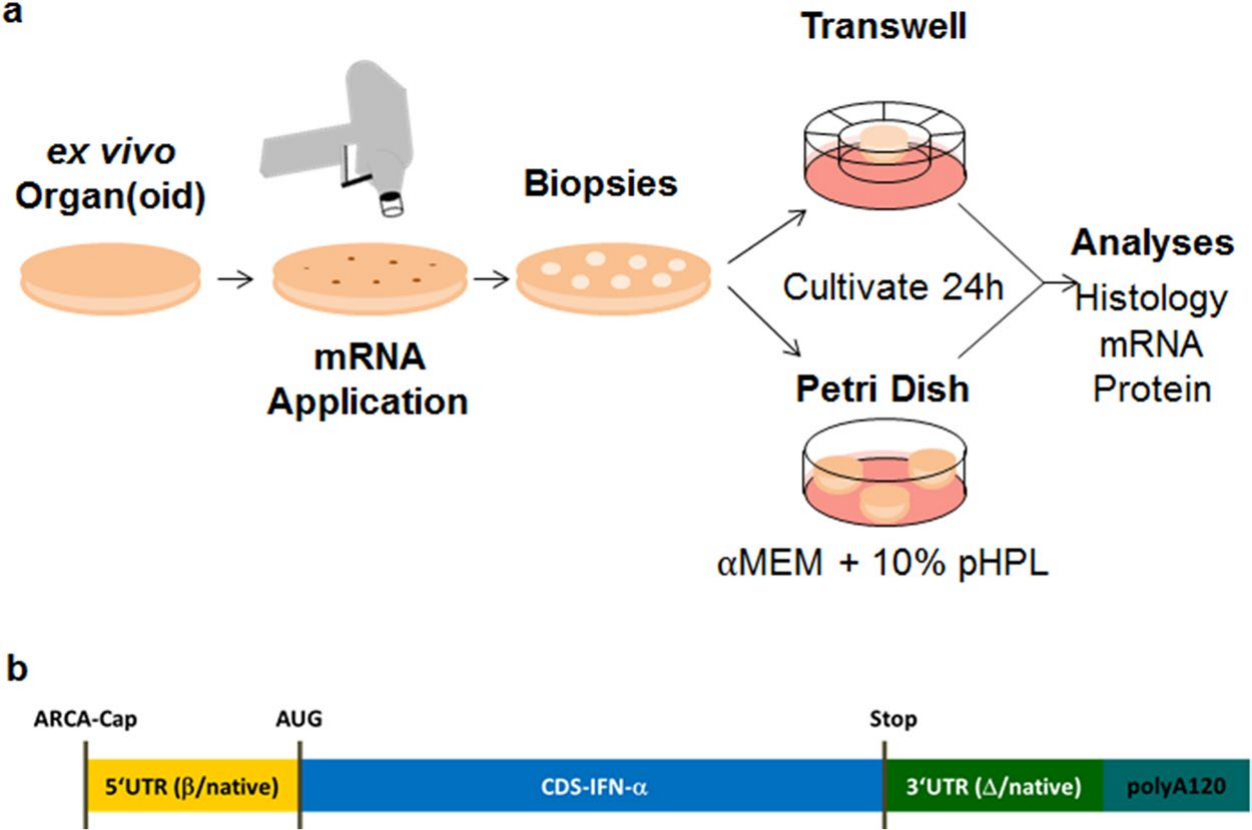
Experimental Setup - biolistic mRNA transfection and mRNA design strategy. (**a**) Skin was transfected *ex vivo* using the BioRad Helios gene gun System with an application pressure of 400 psi. Six mm and eight mm punch biopsies were obtained and explants were cultured for at least 24 h either in transwells or petri dishes, before further analyses. (**b**) Schematic overview of IFN-α coding sequence (for details see also Table 1). Abbreviations: Anti-Reverse Cap Analog (ARCA-Cap); Start Codon (AUG); Stop Codon, (Stop); untranslated region (UTR), 5′ and 3′ end; coding sequence, (CDS); Poly(A) Tail (polyA120).

**Figure 2:**
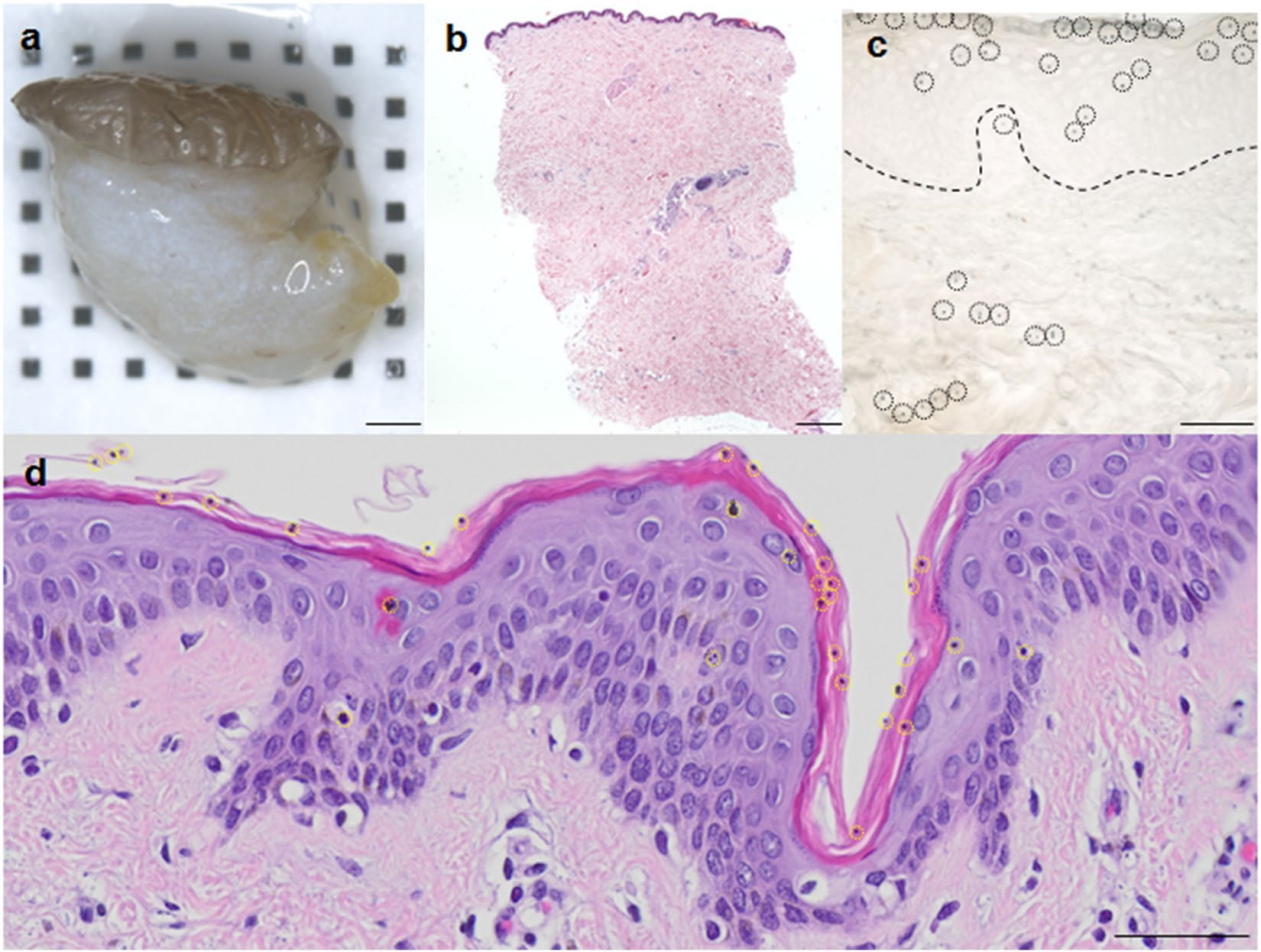
Histology of skin explants. (**a**) Macroscopic picture of a skin biopsy. (**b**) HE staining of a longitudinal section of the biopsy prior to gene gun application (**c**) 10 µm unstained cryo section showing gold particles (indicated by black dotted circles) in the epidermis and dermis of the explant. The dermal-epidermal border was marked by a black dotted line. (**d**) HE staining of a thin 2 µm paraffin section after gene gun application showing 1.6 µm gold particles predominantly in the epidermis (indicated by yellow circles). Scale bar in (**a**,**b**) represents 1000 µm; Scale bars in (**c**,**d**) represent 50 µm.

The general experimental strategy for treating *ex vivo* skin is depicted in Fig. 1a. Next, eGFP mRNA was applied under defined conditions to intact surgically resected skin (Fig. S1b). Eight mm punch biopsies (Fig. 2a) were obtained immediately after biolistic delivery of 1.6 µm diameter gold particles loaded with reference eGFP coding mRNA using a gene gun device. An overview of the sample histology revealed intact skin architecture (Fig. 2). Higher magnification microscopy showed presence of gold particles delivered with 400 psi predominantly in the epidermis. Approximately one third of the gold particles were found within the upper dermal compartment with certain donor variability as visualized in 10 µm thick sections (Fig. 2c). Thin sections (2 µm) performed after gold particle application showed gold particles mainly in the stratum corneum compatible with either local variability or with the notion that thicker (e.g. 10 µm) sections are required to depict distribution of the 1.6 µm diameter particle (Fig. 2d).

**Figure 3:**
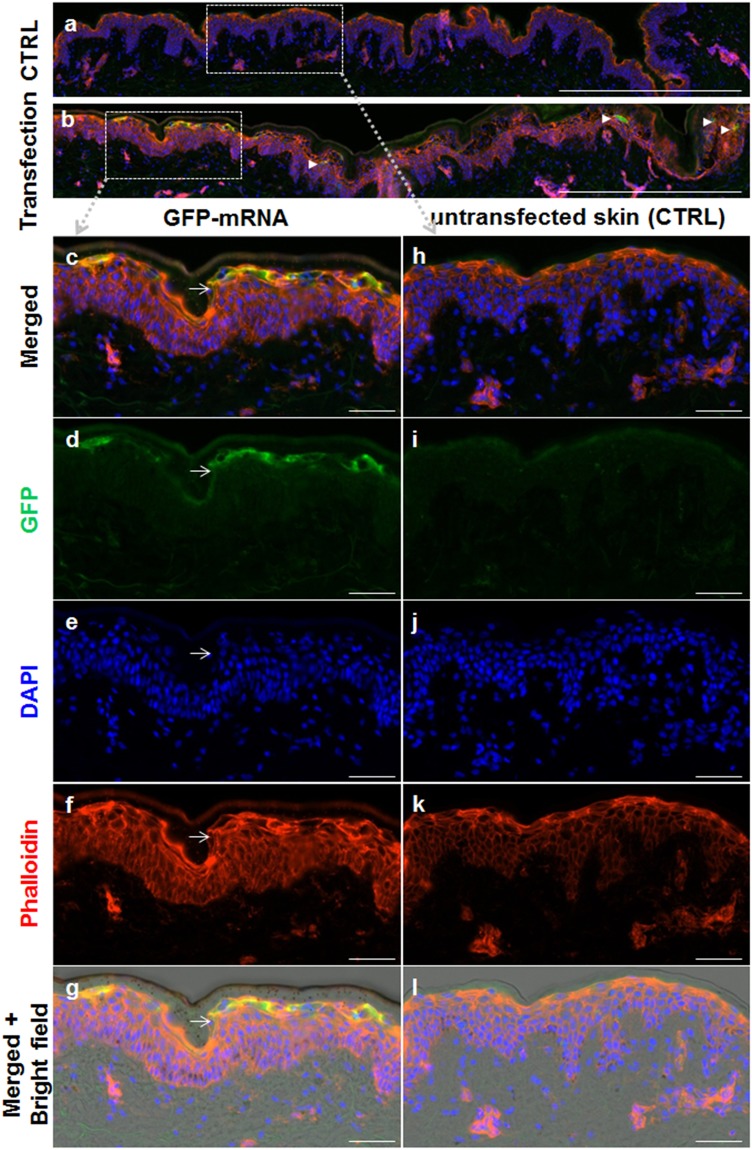
Biolistic eGFP IVT mRNA transfection in human skin explants. (**a**,**h**–**l**) Untransfected control biopsies. (**b**–**g**) Biopsies treated biolistically with 1 μg/μl eGFP mRNA. Reactivity for eGFP (green), F-Actin (red) and nuclei (blue) as indicated. (**b**) Additional suprabasal GFP-transfected cells are marked by white arrowheads. (**g**) Gold particles are visible in merged fluorescence + bright field image after biolistic eGFP mRNA delivery. Scale bars in (a,b) represent 500 μm; Scale bars in (c–l) represent 50 μm.

**Figure 4:**
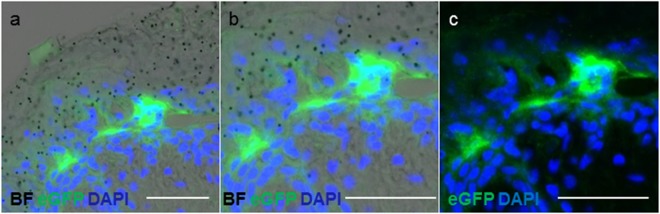
Variable location of eGFP transfected cells. GFP expressing cells were also found in suprabasal position in addition to the superficial position shown in Fig. 3b–g. (**a**) Representative result of suprabasal GFP^+^ epidermal cells 24 h after biolistic transfection, (**b**) in higher magnification, both showing an overlay of GFP and DAPI signals with bright field (BF) to visualize gold microparticles. (**c**) Same magnification than in (**b**) without depicting microparticles. Results from one representative donor (of five tested for GFP transfection) Scale bar 50 µm.

GFP protein expression was determined 24 h after delivery using indirect immunofluorescence with anti-GFP antibody and Alexa488 green fluorescent label revealing predominantly epidermal GFP protein expression in the skin explants. Cytoskeleton and nuclear labeling with phalloidin and DAPI, respectively, additionally allowed confirming intact skin architecture after culture of biolistically treated skin compared to untreated biopsies (Figs 3 and 4). We did not detect eGFP staining after applying the eGFP mRNA gold particles without gene gun/pressure onto the skin. We did also not detect eGFP signal after applying the eGFP mRNA alone without gene gun/gold particles (Fig. S4).

### IFN-α variants differ in their expression level but are comparable between donors

**Figure 5:**
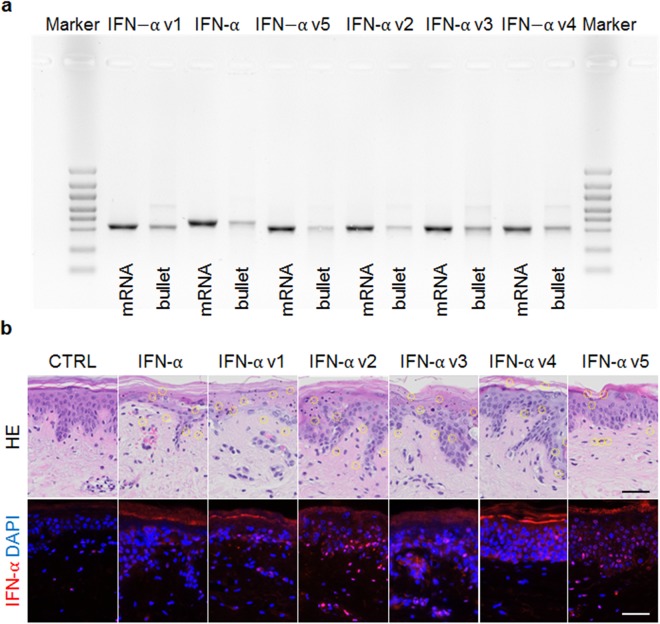
Functionality control of IVT mRNA using biolistic application in *ex vivo* human skin. (**a**) Quality control of mRNA loaded bullets. Samples were analysed in a blinded fashion and thereby the nomenclature is resulting in a mixed order on the gel. Left lane always indicates mRNA before loading onto bullets, right lane shows the mRNA after extracting from loaded bullets. Outer lanes depict size markers. (**b**) Human whole skin explants were transfected using gene gun technology with native IFN-*α* mRNA or five different mRNA variants (for details see Table 1) compared to untransfected explants (CTRL) and subsequently Hematoxylin/Eosin stainings (HE) on paraffin sections were performed. A set of seven representative areas per sample showing the gold particles after biolistic transfection highlighted by yellow circles indicating targeting of mostly epidermis but also dermis. Scale bar represents 50 µm.

**Figure 6:**
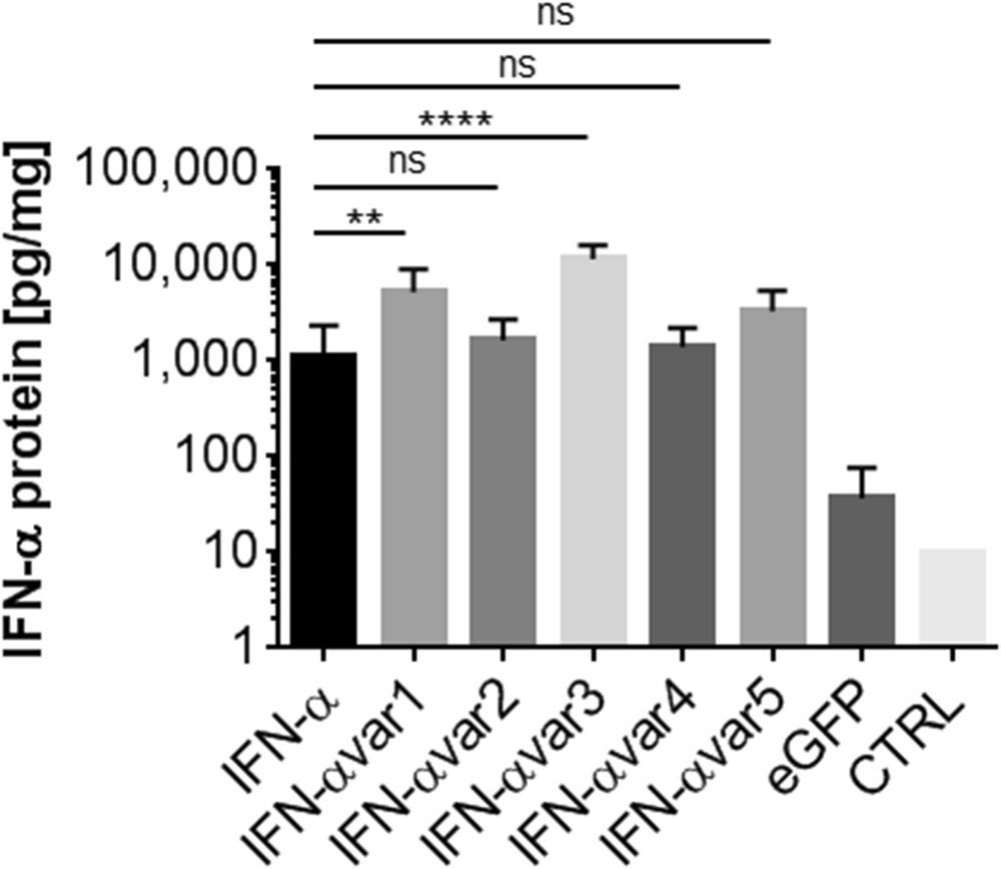
IVT Interferon mRNA variant -based Interferon protein expression in skin explant tissue. Human whole skin explants were transfected using gene gun technology with native IFN-*α* mRNA or five different IFN-α mRNA variants (IFN-α var 1–5, for details see Table 1) compared to eGFP reference transfections and un-transfected control explants (CTRL). IFN-*α* content in explants homogenized 24 h after treatment is shown as pg protein produced per mg skin tissue homogenate. (N = 5; Dunnet’s multiple comparison for the individual variant IFN-α vs. native IFN-α expression; significant differences are indicated ** vs. ****). Data depicted at logarithmic scale.

Next, we generated five differentially modified IFN-α mRNA variants to be compared for their expression efficacy to native human IFN-α mRNA (Fig. 1b and Table 1). Two general types of modifications were applied. In all variants, untranslated regions (UTRs) were exchanged to contain an adapted globin UTR (instead of the IFN UTR). Additionally, in variants 2, 3, 4 and 5, we changed the GC content while considering primarily codons that optimally fit the human translation machinery (as assessed by the codon adaptivity index (CAI)^26^. Bullet loading was measured by PCR clearly indicating space for improvement (Fig. 5a). Protein expression analysis within extracts derived from skin explant tissue that had been exposed to native IFN-α mRNA 24 h earlier by biolistic delivery, revealed efficient expression of IFN-α protein. Variant 1, which differs from the native IFN-α mRNA in the UTR region only, yielded mean 8.5-fold significantly higher expression levels than native human IFN-α mRNA in all donors tested. Out of the 4 variants that were modified in the coding region as well, only variant 3 was found to result in mean ten-fold significantly higher protein expression compared to the native mRNA molecule (Fig. 6). Of note, the expression pattern was consistent among all donors, while the expression levels of the 5 variants differed between the donors tested. Control experiments, based on the same techniques for extract preparation and IFN-α measurement, demonstrated retrieval of 80–90% of human IFN-α protein (Roferon, Roche) injected intradermally into skin explants (Fig. S5). Secreted IFN-α protein measurements in the culture media surrounding the explants from two donors confirmed the results (Fig. S6). Preliminary results of IFN-α secretion time course analysis after a single transfection indicate progressive reduction of IFN-α protein over time (Fig. S7). Interferon mRNA-loaded microparticle distribution was found mostly within the epidermis and protein expression analysis *in situ* was positive for all interferon-transfected samples except for variant three (Fig. 5b).

**Table 1:** Design of IVT Interferon alpha mRNA variants (IFN-*α* var1 – IFN-*α* var5).

IVT mRNA	5′ and 3′ UTR	Sequence variation (%)	GC content (CDS)	Codon optimization
IFN *α*	human IFN *α*	—	48.7	−
IFN *α* var1	modified	—	48.7	−
IFN *α* var2	modified	21.5	49.9	+
IFN *α* var3	modified	21	56.8	+
IFN *α* var4	modified	23	40.4	+
IFN α var 5	modified	21	60	+

IFN-*α* resembels the native human IFN-*α*. All other variants were modified versions of IFN-*α* as indicated. Abbreviations: CDS, coding sequence; UTR, untranslated region. I.

Low levels of IFN-α expression were also detectable in explants after GFP mRNA transfection what may indicate an IFN-α presence in resident cells within skin explants or due to gold particle bombardment and/or GFP expression. A minute IFN-α signal was also detected in untransfected control explants possibly due to surgery or as a response to biopsy explantation. The precise mechanism behind this statistically non-significant low expression has not been addressed in the current study.

## Discussion

Clinical applicability of IVT mRNA for treatments other than vaccination still faces several challenges including, most notably, limited availability of custom-tailored delivery systems and lack of well-tolerated formulations reproducibly allowing for efficient mRNA expression. In addition, immunogenicity concerns are an issue particularly for protein replacement approaches but, less so, for strategies relying on the action of proinflammatory targets such as IFN-α. Here, we showed that IVT IFN-α mRNA can be successfully delivered to human skin by biolistic application. We further demonstated that delivery of equal amounts of two out of five sequence optimized IVT IFN-α mRNA variants resulted in significantly higher temporary IFN-α protein expression compared to the native human IFN-α mRNA across all donors (n = 5) tested. These results provide a proof-of-concept towards treating skin diseases such as NMSCs with mRNA-based therapeutics coding for molecules such as IFN-α. Although the *ex vivo* results obtained here are promising, improved expression over the native sequence would need to be confirmed *in vivo*. Previous IVT mRNA modifications by other groups have shown the potential to improve the intracellular and translational stability of the ensuing variants and their efficiency over the native sequence including modifications to the 5′-cap^27,28^, 5′ and 3′-untranslated regions^29^, the poly (A) tail^30,31^ and codon optimization approaches^32^. Such changes have been shown to result in improved translation, reduced immunogenicity and delayed degradation by nucleases, thereby enhancing the potential for an mRNA to exert a therapeutic effect^33,34^. Using our optimization approach, we found that both exchange of the UTR and modifications of the coding region aiming at an improved and constant CAI but varying the GC content led to significant enhancement in IFN-α expression for some of the variants. This is promising given the fact that the native IFN-α mRNA sequence has already a near optimum codon composition. The results also stress the need to experimentally test the in silico predicted optimizations as not all of the variants turned out to excel the native IFN-α mRNA.

Skin explants derived from human donors or animals represent established models for drug screening^35^. However, as they lack functioning vascular, nervous and immune systems it is not possible to extrapolate *in vivo* responses to the treatment. In addition, there may be variability between donor skin samples (e.g., the age of skin donors tested in this trial was mean = 43.3, range = 27–70 years). As NMSCs primarily affect those in mid-life or later, it is possible that the response observed for younger skin might differ from the one of the skin of the target population for the planned treatment.

The biolistic approach utilized here uses mRNA-loaded gold particle bombardment to deliver IVT mRNA through the stratum corneum to the epidermis. Although this approach has shown great promise in DNA vaccination^36^, it has several limitations. The entry of the gene-gunned gold particles can cause cell damage to the surrounding tissue, which may complicate the interpretation of *in vivo* results. Alternative technology like microinjection^37^ or needle-free injection^38^ may offer certain advantages over particle-based approaches including the possibility to also target the dermis. In addition, the gene gun technique is not compatible with testing the impact of different transfection reagent formulations which have been shown to enhance the uptake of mRNA as well as facilitating endosomal escape and boosting *in vivo* potency (reviewed in^39^). Further limitations of particle-based transfection technology relate to a still limited efficiency of particle loading. In addition, in our study, we observed rapid decline of IFN-α protein secretion after one single transfection. Whereas temporary expression of the therapeutic molecule can be one goal of IVT mRNA-based therapy several aspects requiring further improvement could be identified in this study.

Therefore, focusing on alternative strategies that employ optimized transfection reagent formulations in particle-free systems would enable us to reduce the required dose of IVT mRNA needed to produce a therapeutic effect. Our future work will also be focused on identifying a suitable formulation for clinical development for sensitive areas of the skin, such as face, where a gene gun approach would not be suitable.

## Materials and Methods

### Preparation of RNA bullets for the gene gun system

RNA bullets were prepared by mixing 25 mg gold microcarriers (1.6 µm diameter; Bio-Rad; 1652264) with 400 µl 2-propanol and sonicated for 5 minutes. RNA solution (200 µl) was prepared containing 100 µg of RNA (eGFP-mRNA; 1 µg/µl; AmpTec; 7001-M0514/1000), 0.5 M ammonium acetate and nuclease-free H_2_0 and added drop wise to the gold microcarriers while continuously vortexing the mixture. RNA was precipitated by incubating the mixture at −20 °C for at least one hour. Thereafter the RNA-gold complexes were washed three-to-four times in 100% dried ethanol and finally resuspended in 3 ml 100% ethanol supplemented with 0.04 mg/ml polyvinylpyrrolindone (PVP). The suspension was coated onto the inner wall of tefzel tubing (3.175 mm outer diameter; 2.36 mm inner diameter; #1652441, Bio-Rad) according to the manufacturer’s protocol and cut into approximately 40 cartridges (approximately 15 mm length; tubing cutter #1652422; Bio-Rad) which were stored at −20 °C until use for a maximum of eight weeks.

### Biolistic transfection of human skin explants

Human full thickness skin was obtained from biologic waste material after informed consent as approved by the ethical committee of the region of Salzburg (vote number: 415-E/1990/8–216). Skin was wiped with sterile PBS to remove adherent blood after placing in a laminar air hood (Fig. S1). For transfection the handheld Bio-Rad Helios gene gun system was loaded with eGFP-mRNA coated gold particles using a helium gas pressure of 400 psi at a distance of 2.5 cm. After transfection six vs. eight mm diameter punch biopsies were obtained and maintained in α-MEM cell culture medium supplemented with 2.5 mL Dipeptiven (Fresenius Kabi) and 10% pooled human platelet lysate (pHPL) as described previously^40^ (at the air-liquid interface in a humidified air incubator at 5% CO_2_ for up to five days. Subsequently the biopsies were either snap-frozen in liquid nitrogen, or transferred to RNA*later* Solution (Thermo Fisher Scientific; AM7020) for RNA and protein analysis or fixed in 4% paraformaldehyde over night at 4 °C before further histology processing as described previously^41^. Control experiments were performed testing the application of eGFP mRNA-loaded gold particles without gun/pressure as well as application of eGFP mRNA alone. Untreated skin biopsies were cultivated for 24 h and subsequently paraffin sections were performed and stained for eGFP/Phalloidin/DAPI and hematoxylin/eosin. The eGFP mRNA loaded gold particles were applied onto a transparent film dressing (Tegaderm; 1624W; 3 M Health Care, USA) using the gene gun application. Subsequently the film patch including the mRNA loaded gold particles was placed onto the *ex vivo* skin and 8 mm biopsies were taken and cultured for 24 h. Subsequently paraffin sections were performed and stained for eGFP (anti-GFP; chicken IgY; Life Technologies; A10262) Phalloidin (Alexa Fluor 568, A12380; Life Technologies, USA) and DAPI and hematoxylin and eosin. In addition 1 µg eGFP mRNA in PBS was applied onto the *ex vivo* skin. The droplet was incubated for about 30 minutes and subsequently covered with a transparent film dressing (Tegaderm). Skin biopsies were taken and cultivated for 24 h and paraffin sections were performed and stained as indicated (Fig. S4).

### Immunohistochemistry, TUNEL labeling and histochemistry

Ten µm cryosections were performed for fluorescence analysis and processed essentially as described previously^42^. GFP antibody (anti-GFP; chicken IgY; Life Technologies; A10262) was used at 1:500 dilution and detected by Alexa Fluor 488-conjugated donkey anti-chicken IgY antibody (1:500; Dianova 703-545-155). The cytoskeleton was detected by staining F-actin using Alexa Fluor 568 Phalloidin (Life Technologies; A12380; 1:400) and slides were mounted in Roti-mount Fluor Care DAPI (Carl Roth; HP20.1) containing DAPI (4′,6-Diamidin-2-phenylindol) for counterstaining. TUNEL labeling was performed following manufacturer’s instructions (Promega Dead End Colorimetric TUNEL System #G7130). Hematoxylin and eosin staining was done in the linear slide stainer (Leica ST4040) in two µm sections of paraffin embedded tissue using Mayer’s Hemalum (MERCK; 1.09249.2500) and Eosin Y (MERCK; 1.15935.0100).

### Image acquisition

Slides were scanned automatically in 20x or 40x magnification using the VS-120-L Olympus slide scanner 100-W system. Pictures were processed using the Olympus VS-ASW-L100 program and figures were prepared using Microsoft PowerPoint 2010.

### Preparation of *in vitro* transcribed mRNA

*In vitro* transcribed mRNA for eGFP (1 µg/µl; 7001-M0154/1000) was obtained from AmpTec, Germany. Native human IFN-α mRNA, with regard to the coding DNA sequence (CDS) and the flanking UTRs, as well as five sequence-optimized variants of human IFN-α were synthesised by Eurofins, Germany. *In vitro* transcription and purification of IFN-α mRNA variants was performed by AmpTec, Germany, according to published procedures^43^. Briefly, RNA was transcribed using T7 RNA polymerase and ARCA-NTP-Mix from a linear template generated by PCR. Purified mRNA was treated with antarctic phosphatase and re-purified. RNA integrity and purity was controlled by analysis on an Agilent Bioanalyzer 2100 and RNA 6000 Nano Kit (Agilent, USA). The RNA concentration was determined by spectrophotometry on an Infinite 200 PRO multimode reader (Tecan AG, Switzerland).

### Analysis of human IFN-α protein

Analysis of mRNA transfected skin explant tissue was done using two approaches: (i) we either subjected supernatants of tissue cultures or (ii) protein extracts derived from explants to IFN-α measurement by ELISA. Protein was extracted using cell extraction buffer (10 mM HEPES, 10 mM KCl, 0.1 µM EDTA, 0.3% NP40 and Roche protease inhibitor, according to manufacturer’s protocol). Measurement of human IFN-α was performed using the human IFN-α (subtype 2, IFN-α2) ELISA development kit (MABTECH AB, Sweden, according to manufacturer instructions). Protein concentration of extracts was determined using a conventional BCA test (Pierce, USA; according to manufacturer’s protocol). IFN-α and protein measurements were taken on an Infinite 200 PRO multimode reader (Tecan AG, Switzerland).

### Ethics Statement

Human full thickness skin was obtained from biologic waste material after informed consent as approved by the ethical committee of the region of Salzburg (vote number: 415-E/1990/8–216). Skin samples were collected in accordance with the Declaration of Helsinki after written informed consent from healthy volunteers.

## Electronic supplementary material

Supplementary Data

## Data Availability

All data produced or analyzed during this study are included in this published article and its Supplementary Information files.
