# Supplementary material for: Evaluation of modified Interferon alpha mRNA constructs for the treatment of non-melanoma skin cancer
Source: Sci Rep. 2018 Aug 28;8:12954. doi: 10.1038/s41598-018-31061-w (PMC6113332; doi:10.1038/s41598-018-31061-w)
Supplement: Supplementary file 1 — Supplementary Data [file 41598_2018_31061_MOESM1_ESM.doc]

Supplementary Information

**Evaluation of modified Interferon alpha mRNA constructs for the treatment of non-melanoma skin cancer**

Sarah Hochmann,^1,2^ Michaela Mittermeir,^1,2^ Radmila Santic,^3^ Frieder Koszik,^3^ Lanay Griessner,^3^ Alina Sarah Sonderegger,^1,2^ Thomas Hoffmann,^3^ Elisabeth Russe,^4^ Sandra Scheiblhofer,^5^ Richard Weiss,^5^ Markus Mandler,^3^ Achim Schneeberger,^3^ and Dirk Strunk^1,2^,*

^1^Cell Therapy Institute, Paracelsus Medical University, Salzburg, Austria; ^2^Spinal Cord Injury and Tissue Regeneration Center Salzburg (Sci-TReCS), Paracelsus Medical University, Salzburg, Austria; ^3^Accanis Biotech F&E GmbH & Co KG, Vienna Biocenter, Austria; ^4^Department of Plastic, Aesthetic and Reconstructive Surgery, Hospital Barmherzige Brueder, Salzburg, Austria; ^5^Department of Molecular Biology, Paris Lodron University, Salzburg, Austria

Corresponding author mail: [dirk.strunk@pmu.ac.at](mailto:dirk.strunk@pmu.ac.at)

Keywords: mRNA, Gene gun, skin explant, gene therapy, Interferon


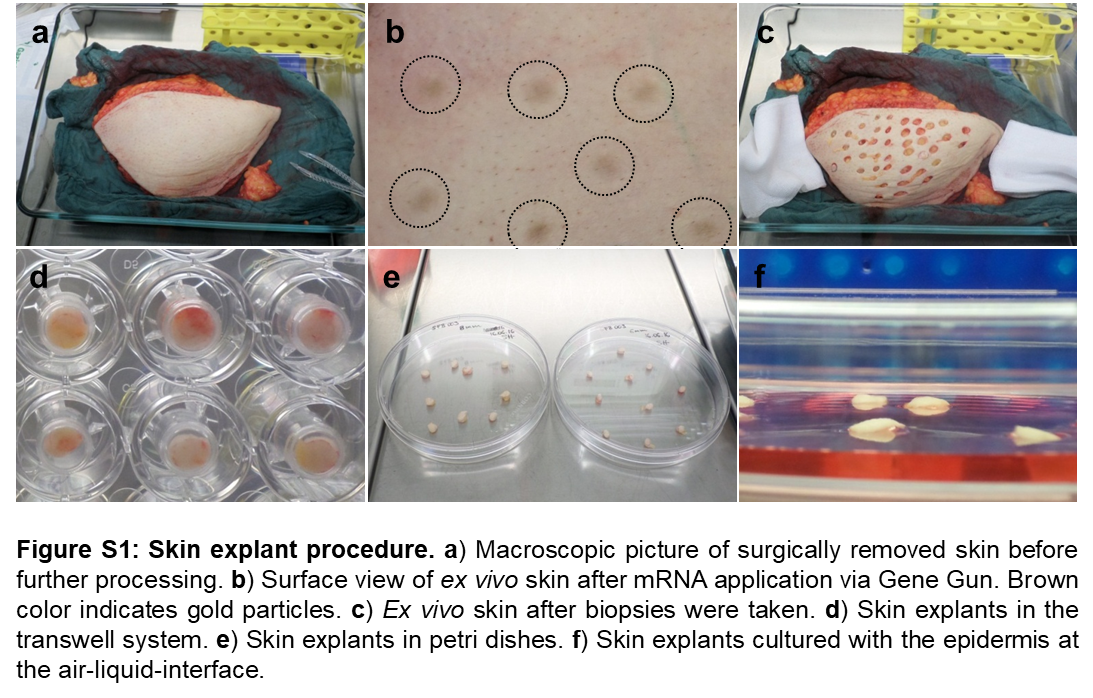
**Figure S1: Skin explant procedure.** a) Macroscopic picture of surgically removed skin before further processing. b) Surface view of ex vivo skin after mRNA application via gene gun. Brown color indicated gold particles. c) Ex vivo skin after biopsies were taken. d) Skin explants in the transwell system. e) Skin explants in petri dishes. f) Skin explants cultured with the epidermis at the air-liquid-interface.


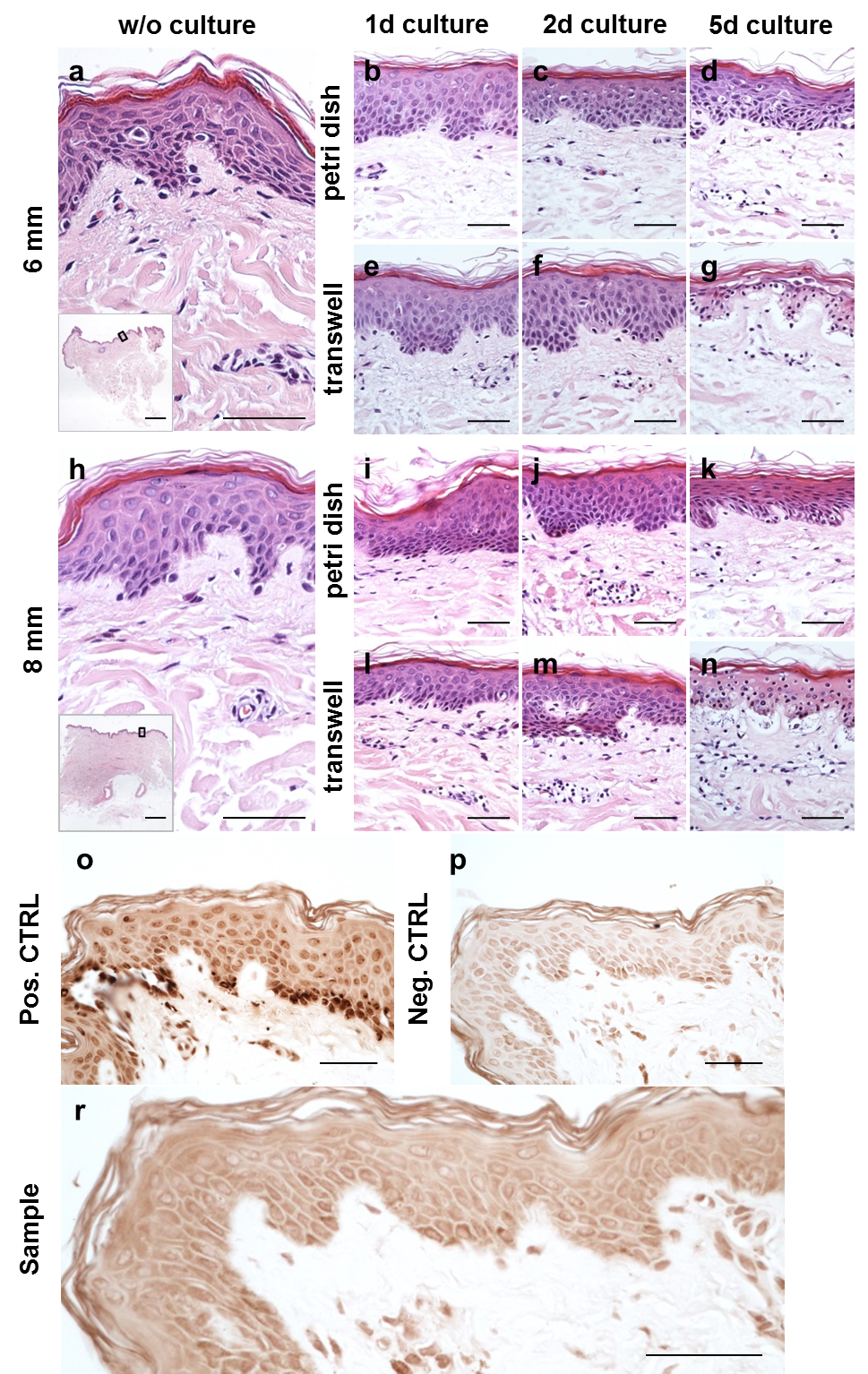


**Figure S2: Human skin explant survival timecourse of 6 mm and 8 mm punch biopsies in petri dish and transwell cultures.** a-g) 6 mm skin biopsies. a) Skin was fixed in 4% PFA directly after surgery. b-d) Sin was cultured in a petri dish for b) one day c) for two days and d) for five days respectively. e-f) Skin was cultured in transwells for e) one day, f) two days and g) five days respectively. h-n) 8 mm skin biopsies. h) Skin was fixed in 4% PFA directly after surgery. i-k) Skin was cultured in petri dishes for i) one day, j) for two days and k) for five days respectively. l-n) Skin was cultured in transwells for l) one day, m) two days and n) five days respectively. Scale bars in inserts represents 1000 µm, all other scale bars represent 50 µm. o-r) Minimum signs of apotosis were found after explant culture using TUNEL stain at day 5. Positive and negative control (Pos. / Neg. CTRL) following manufacturer’s instruction; dark brown nuclei indicate positive staining, i.e. apoptosis. One representative sample is shown.


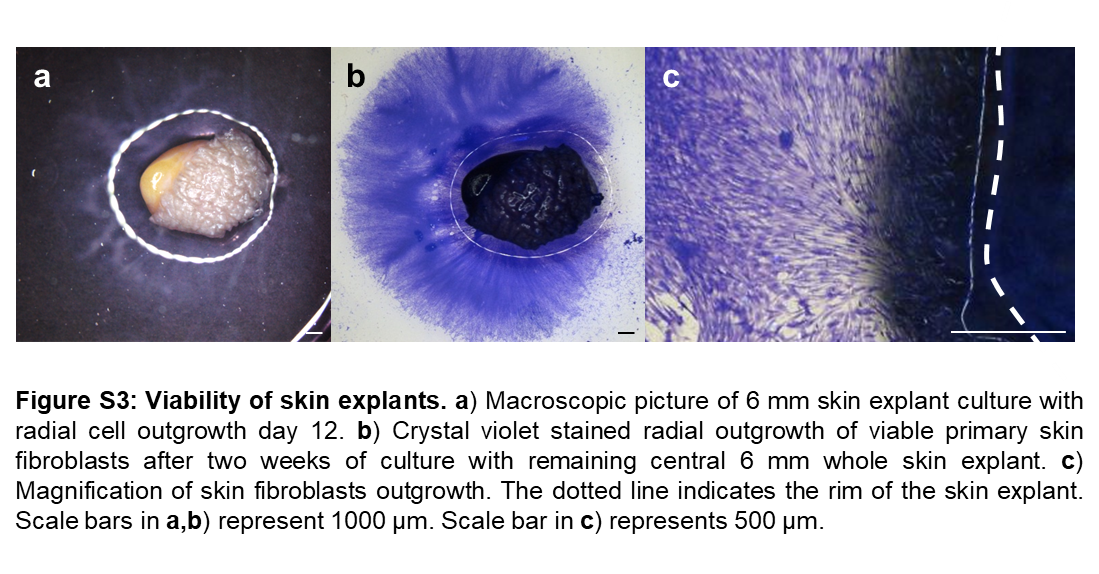


**Figure S3: Viability of skin explants.** a) Macroscopic picture of 6 mm skin explant culture with radial cell outgrowth day 12. b) Crystal violet stained radial outgrowth of viable primary skin fibroblasts after two weeks of culture with remaining central 6 mm whole skin explant. c) Magnification of skin fibroblasts outgrowth. The dotted line indicated the rim of the skin explant. Scale bars in a,b) represent 1000 µm. Scale bar in c) represents 500 µm.

| 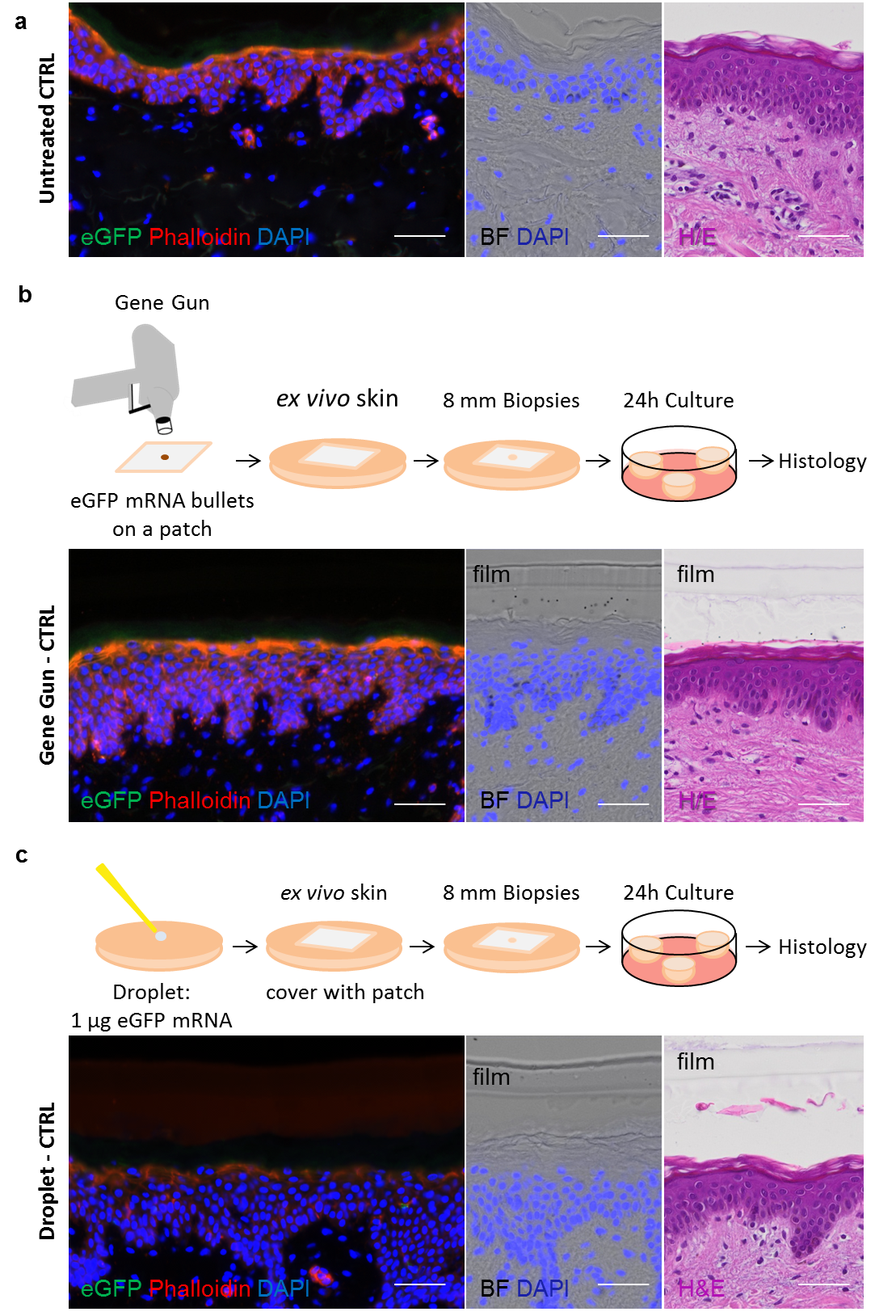 |
| --- |
|  |

**Figure S4: Gene gun control experiments**. a) Un-transfected control biopsies. b, c) Control biopsies that were treated as illustrated graphically with b) non-pressurized application of eGFP mRNA-loaded gold particles on a Tegaderm transparent film dressing (to address the reviewer question if transfection occurs when mRNA loaded gold particles were placed onto the skin without pressure) and c) addition of 1 µg eGFP mRNA in PBS onto the ex vivo skin to control if free mRNA results in transfection. (left pictures) Reactivity for eGFP (green), Phalloidin (red) and nuclei (blue) as indicated. (middle pictures) Bright field/DAPI overlay pictures to visualize the Tegaderm film b, c) that covers the gold microparticles (in b; no microparticles visible in c). Larger dark spots in the epidermis represent melanin. Right pictures show hematoxylin/eosin (H/E) stains illustrating intact skin morphology. Scale bars represent 50 μm.


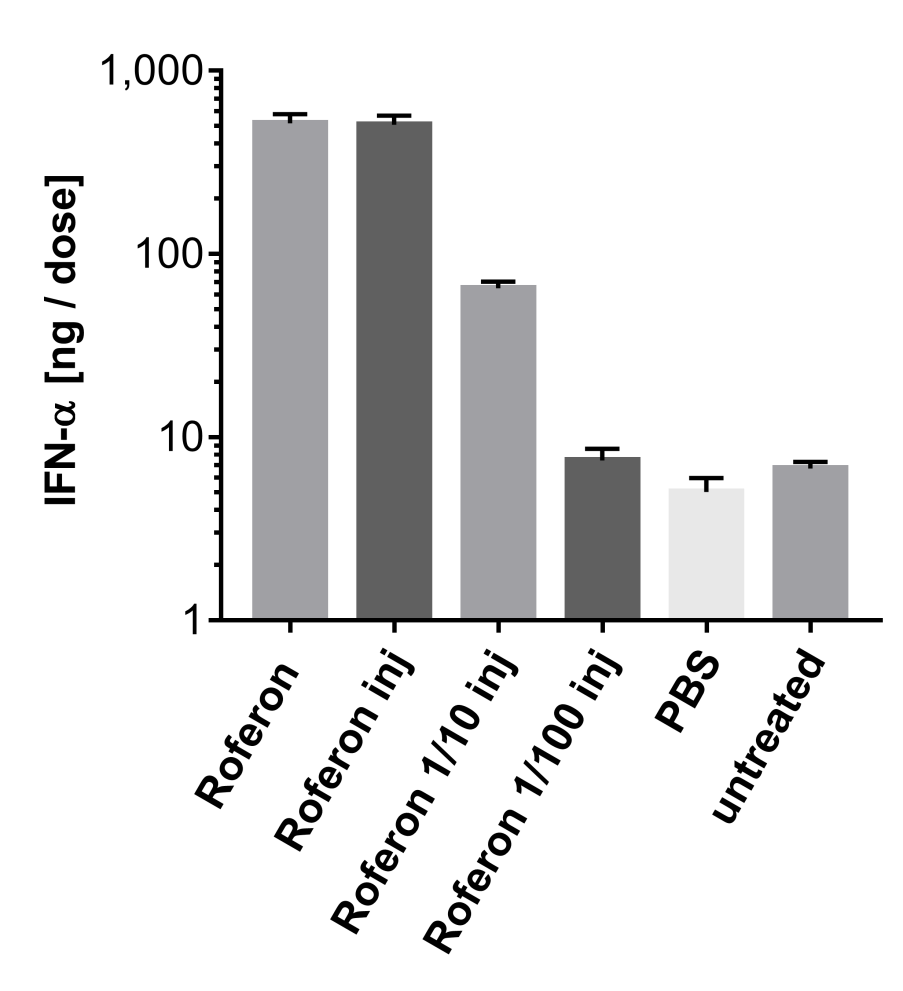


**Figure S5: Interferon alpha protein injection recovery.** To determine the recovery of interferon alpha protein from skin explant biopsies we injected clinically used IFN-
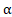
 (Roferon-A, 0.01 mL per Injection; 4.5 Mio. I.U./0.5 mL; Roche) either pure (Roferon inj) or after 1/10 or 1/100 dilution in PBS in one pilot experiment. ELISA analysis was done as described in the methods section comparing IFN-
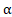
 content in Roferon-A solution to values obtained of protein lysates from skin biopsies after Roferon-A injection, PBS injection or from untreated skin. Comparison of IFN-α values in Roferon-A and in protein lysates of Roferon-A injected biopsies revealed a high recovery in biopsies (>90%) and did not show a statistical significant difference (p=0,567; Roferon-A: 517,5 ± 9,592 ng/dose; n=40; Roferon-A injected biopsy: 507,1 ± 15,4 ng/dose, n=16; values: mean ± SEM; statistical analysis: one-way ANOVA and unpaired t-test comparing Roferon-A to Roferon-A injected samples, respectively).

***
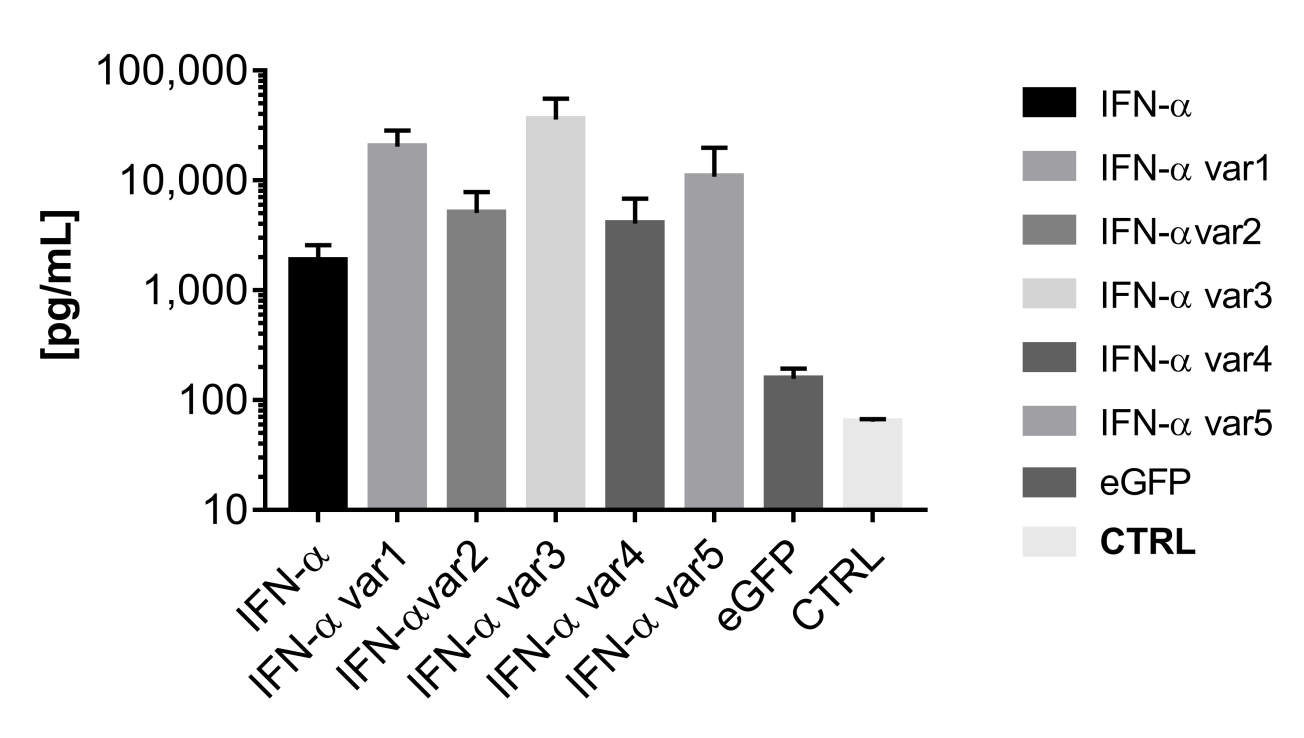
***

**Figure S6: IVT Interferon mRNA variant -based Interferon protein secretion from skin explant tissue.** Human whole skin explants were transfected using gene gun technology with native IFN
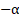
 mRNA or five different IFN-α mRNA variants (IFN-α var 1 – 5, for details see Table 1) compared to eGFP reference transfections (eGFP) and un-transfected control explants (CTRL). IFN
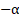
 content in media harvested 24 h after treatment is shown as pg protein produced per mL medium. (N = 2 donors / IVT mRNA).


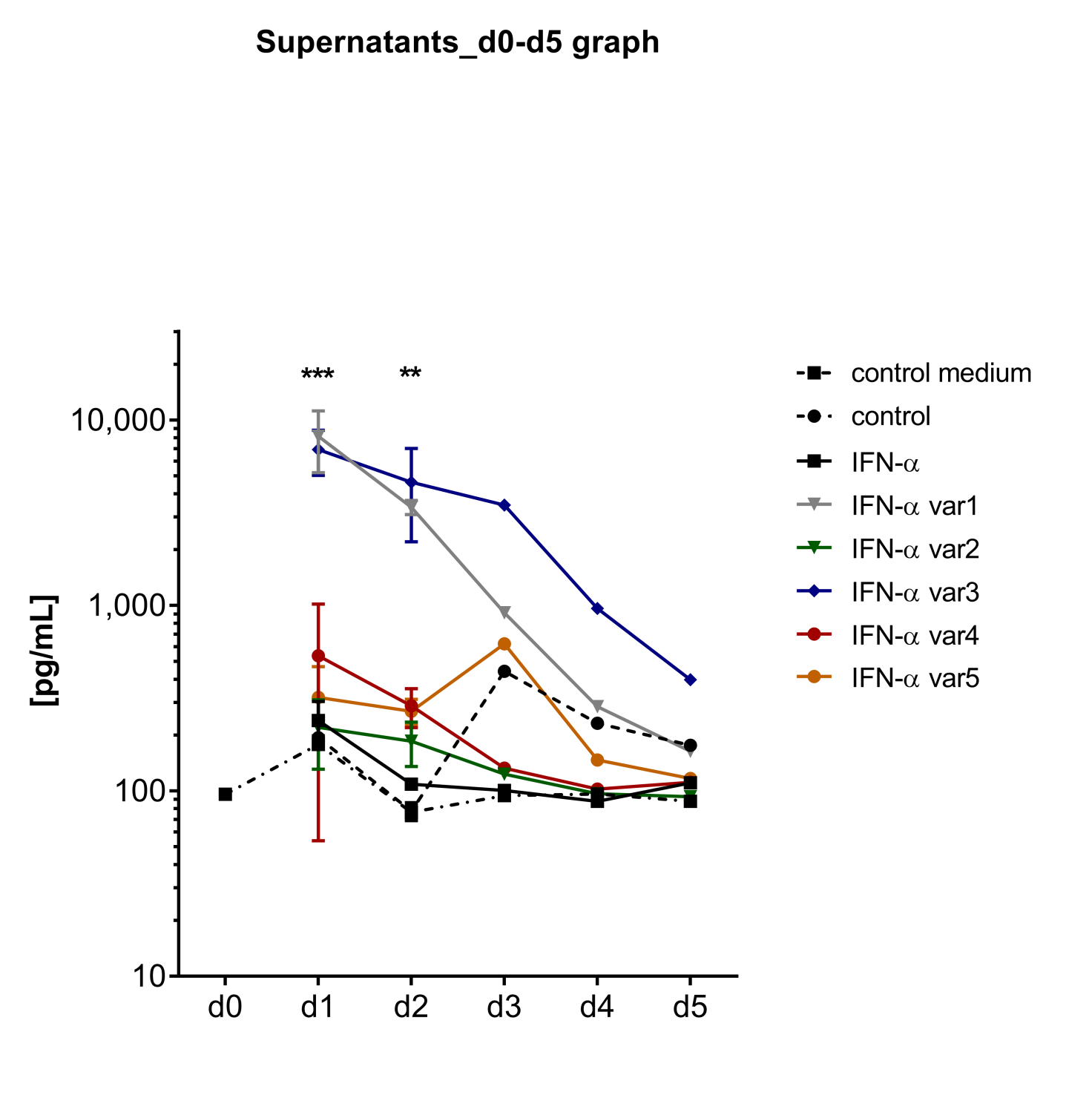


**Figure S7: Time course of IVT Interferon mRNA variant -based Interferon protein secretion around skin explant tissue.** Human whole skin explants were transfected as described in Fig. S6 compared to un-transfected control skin sample medium (control) and empty medium (control medium). IFN
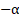
 content in media harvested before and at indicated time after transfection is shown as pg/mL. Statistical analysis (one-way Anova and Tukey's multiple comparisons test revealed highly significant increase in IFN-α secretion for variants 1 and 3 as compared to the remaining groups at 24h post transfection (***p<0.0001). Variants 1 and 3 are not significantly different. At 48h, Variant 3 is significantly higher than the remaining groups (**p ≤ 0.013). Again variants 1 and 3 are not significantly different.


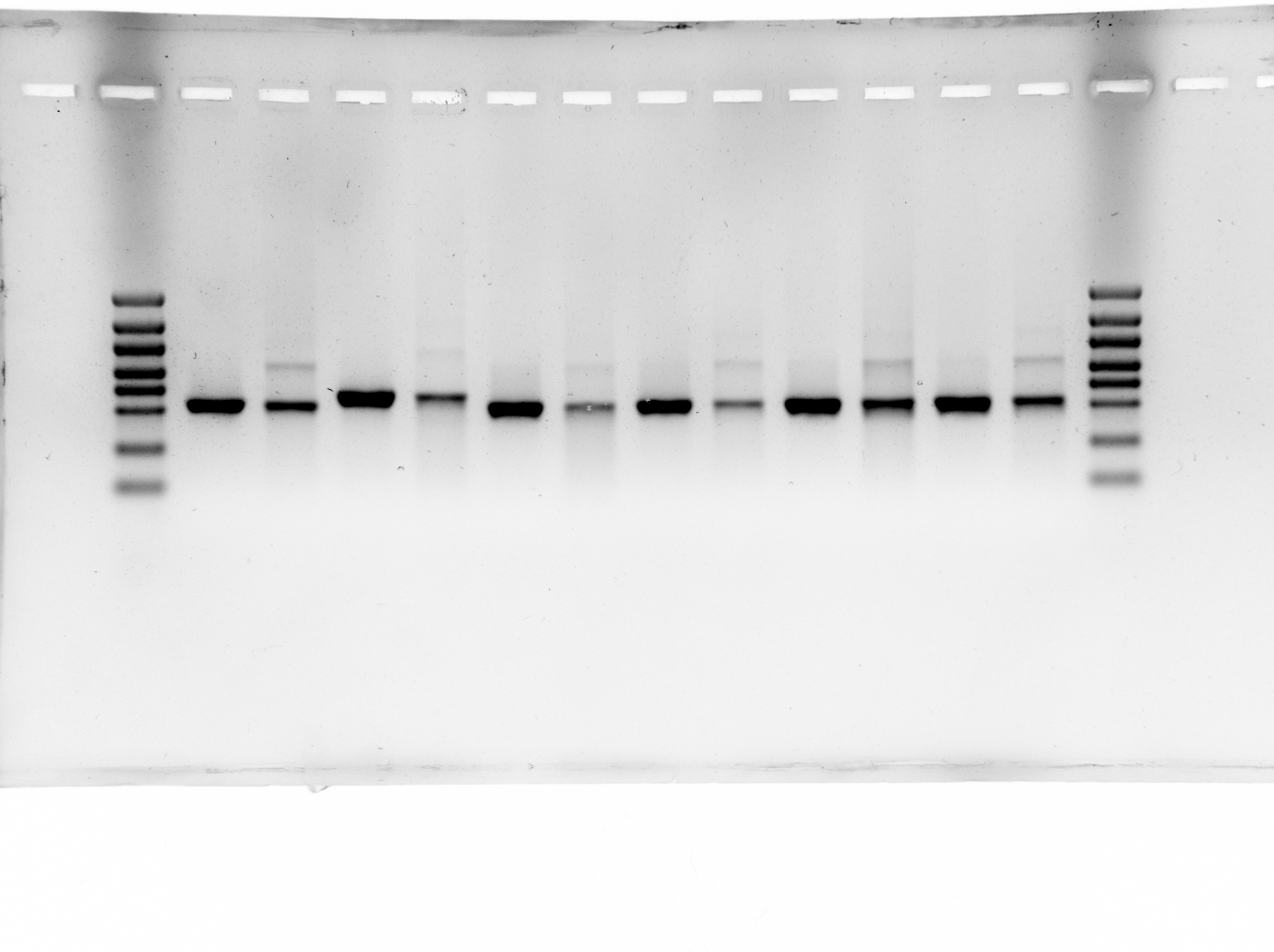


**Figure S8: Original Photograph depicting the original full length gel shown in Figure 5.** Following the digital image and integrity policies of The Journal this original photograph is depicted. The gel was recorded on a ChemiDoc XRS+ system using Image Lab 4.0.1 software (Biorad) using the “auto-exposure“ tool to get optimized exposure time without saturated pixels. Subsequently, contrast as shown in Figure 5 was optimized using the “auto contrast” feature of Adobe Photoshop 7.0.
